# Shortening cardioplegic arrest time in patients undergoing combined coronary and valve surgery: results from a multicentre randomized controlled trial: the SCAT trial

**DOI:** 10.1093/ejcts/ezx087

**Published:** 2017-04-24

**Authors:** Chris A. Rogers, Radek Capoun, Lauren J. Scott, Jodi Taylor, Anil Jain, Gianni D. Angelini, Pradeep Narayan, M-Saadeh Suleiman, Kunal Sarkar, Raimondo Ascione

**Affiliations:** a Clinical Trials and Evaluation Unit, School of Clinical Sciences, University of Bristol, Bristol, UK; b Bristol Heart Institute, School of Clinical Sciences, University of Bristol, Bristol, UK; c SAL Hospital and Medical Institute, Ahmedabad, India; d Rabindranath Tagore International Institute of Cardiac Sciences (RTIICS), Kolkata, India

**Keywords:** Myocardial protection, Beating heart coronary surgery, Cardioplegic arrest, Valve surgery

## Abstract

**OBJECTIVES:** Combined coronary artery bypass grafting and valve surgery requires a prolonged period of cardioplegic arrest (CA) predisposing to myocardial injury and postoperative cardiac-specific complications. The aim of this trial was to reduce the CA time in patients undergoing combined coronary artery bypass grafting and valve surgery and assess if this was associated with less myocardial injury and related complications.

**METHODS:** Participants were randomized to (i) coronary artery bypass grafting performed on the beating heart with cardiopulmonary bypass support followed by CA for the valve procedure (hybrid) or (ii) both procedures under CA (conventional). To assess complications related to myocardial injury, we used the composite of death, myocardial infarction, arrhythmia, need for pacing or inotropes for >12 h. To assess myocardial injury, we used serial plasma troponin T and markers of metabolic stress in myocardial biopsies.

**RESULTS:** Hundred and sixty patients (80 hybrid and 80 conventional) were randomized. Mean age was 66.5 years and 74% were male. Valve procedures included aortic (61.8%) and mitral (33.1%) alone or in combination (5.1%). CA time was 16% lower in the hybrid group [median 98 vs 89 min, geometric mean ratio (GMR) 0.84, 95% confidence interval (CI) 0.77–0.93, *P* = 0.0004]. Complications related to myocardial injury occurred in 131/160 patients (64/80 conventional, 67/80 hybrid), odds ratio 1.24, 95% CI 0.54–2.86, *P* = 0.61. Release of troponin T was similar between groups (GMR 1.04, 95% CI 0.87–1.24, *P* = 0.68). Adenosine monophosphate was 28% lower in the hybrid group (GMR 0.72, 95% CI 0.51–1.02, *P* = 0.056).

**CONCLUSIONS:** The hybrid procedure reduced the CA time but myocardial injury outcomes were not superior to conventional approach.

**TRIAL REGISTRATION:** ISRCTN65770930.

## INTRODUCTION

The number of patients requiring surgery to treat concomitant valve and coronary disease is rapidly increasing [1–3]. Between 2001 and 2010, the proportion of patients requiring valve procedures, combined valve and coronary artery bypass grafting and other procedures increased from 29% to 50% [2, 3]. Combined valve and coronary artery bypass grafting surgery leads to marked myocardial injury and is associated with in-hospital mortality twice that of valve surgery alone, and 4 times that of coronary artery bypass grafting alone [4–6]. Determinants of myocardial injury include prolonged cardioplegic arrest (CA) and cardiopulmonary bypass (CPB) times, and concomitant ischaemic and hypertrophic disease. Cardiac-specific complications associated with myocardial injury includes death, myocardial infarction (MI), arrhythmias, need for pacing, need for inotropes and low-cardiac output syndrome [7–15]. The presence of left ventricular hypertrophy secondary to aortic stenosis increases susceptibility to ischaemia with accelerated loss of high-energy nucleotides and greater accumulation of lactate [11–16]. Myocardial injury in these patients is exacerbated by the concomitant presence of coronary disease, left ventricular hypertrophy and prolonged CA time [7, 16, 17]. In a small pilot study in 40 patients (unpublished data) with coronary and valve disease, we used our expertize in beating heart coronary surgery [17–21] and our previous experience in on-pump beating heart coronary surgery [22] to explore the feasibility of shortening CA time with a view to reduce myocardial injury. We performed coronary surgery first with CPB support on the beating heart, followed by CA for the valve procedure (hybrid procedure) and observed a shorter CA time and a trend to less MI, arrhythmias, need for pacing or for inotropes. The aim of this trial was to ascertain the safety and efficacy of the hybrid procedure in reducing myocardial injury as assessed by a composite of related postoperative cardiac-specific complications and by release of troponin T and markers of myocardial oxidative stress in biopsies.

## MATERIALS AND METHODS

### Study design

A multicentre parallel-group randomized controlled trial (ISRCTN 65770930).

### Participants

Adults aged between 16 and 85 years with severe coronary disease and aortic and/or mitral valve disease (confirmed by transthoracic echocardiography and angiogram) were eligible. History of diabetes, malignancy, debilitating neurological disease, ongoing sepsis or endocarditis, or preoperative creatinine >160 µm/l, or need for emergency or salvage procedures were exclusion criteria.

### Study settings

The study was conducted at the Bristol Heart Institute, Bristol (UK), Rabindranath Tagore International Institute, Kolkata and the SAL Hospital, Ahmedabad (India) by surgeons willing to operate using either methods. The University Hospital Bristol NHS Foundation Trust sponsored the trial in the UK. In India, the study was sponsored by the 2 host institutions. The trial was approved by the Southmead Research Ethics Committee (ref. CS/2006/2267) in the UK and by local hospital Ethics Committees in India.

### Interventions

Participants were randomly allocated to either (i) *conventional surgery* with both coronary and valve surgery being performed during CPB and CA; or (ii) *hybrid surgery* with coronary surgery carried out first on the beating heart with CPB support, followed by CA to undertake valve surgery.

### Surgical methods

Surgery, anaesthesia and postoperative management were according to standardized protocols [7, 16]. Moderate hypothermic CPB (32°C) was used. For the hybrid group on-pump beating heart coronary grafting was carried out at CPB mean arterial pressure around 75 mmHg to optimize myocardial perfusion. Mode of cardiac stabilization and coronary grafting was according to established routine at each centre. For both groups, CA was achieved with cold (4–6°C) blood cardioplegia: patient’s blood and St Thomas’ I cardioplegic solution (4:1) with extra K^+^ and Mg^2+^ added. Final K^+^ and Mg^2+^ concentrations were 20 and 4 mM, respectively. Antegrade cardioplegia was delivered under pressure (100–150 mmHg) directly into the coronary ostia or the aortic root, while retrograde cardioplegia was delivered under pressure (20–30 mmHg) directly into the coronary sinus at 20 min intervals. Following surgery, patients were admitted to the cardiac intensive care unit and managed by blind intensivists. Decisions regarding need for inotropic support, pacing and mode of ventilation were based on established routine clinical care [7, 18–22]. Heart rate, rhythm and ST changes were continuously monitored during the first 72 h postoperatively. A twelve-lead electrocardiogram (ECG) was performed prior to surgery, at 4 h postoperatively, and then daily for 5 days.

### Outcome measures

#### Primary outcome: composite of complications related to myocardial injury

This included in-hospital death (in-hospital or 30-day death), postoperative MI (new Q waves ≥0.04 cm or a reduction in R waves of >25% in at least 2 leads), arrhythmia (supraventricular tachycardia/atrial fibrillation or ventricular tachycardia/ventricular fibrillation, need for cardiac pacing >12 h, need for inotropic support >12 h (dobutamine ≥3 µg/kg/min, and/or adrenaline ≥1 µg/kg/min, and/or dopamine ≥5 µg/kg/min or any dose of enoximone).

#### Secondary outcomes: direct myocardial injury

This was assessed by measuring serial plasma release of cardiac troponin T (cTnT) at baseline (prior to surgery), and at 1, 4, 12, 24, 48 and 72 h postoperatively. In addition, markers of metabolic stress were measured in a consecutive series of patients in Bristol in myocardial biopsies collected from the apex of the left ventricle using a Trucut biopsy needle 5 min following CPB institution, (control biopsy), and 10 min after releasing the cross-clamp (reperfusion biopsy). Specimens were immediately frozen in liquid nitrogen and stored until processing [7].

### Other clinical outcome

We recorded duration of CPB and CA time, low-cardiac output (defined as need for adrenaline, dobutamine, enoximone or dopamine (dose ≥ 5ug/kg/min) or intra-aortic balloon pump (IABP) for >3 h), blood loss, transfusion requirement, intubation time, pulmonary, infective, renal, gastrointestinal (GI) and neurological complications, cardiac intensive care unit and hospital stay.

### Sample size

The calculation was based on the incidence of cardiac complications related to myocardial injury observed is the pilot study including death, MI, arrhythmias, need for pacing and need for inotropic support for >12 h. The pilot showed that 50% of patients in the hybrid group experienced this composite compared to 80% of patients in the conventional group i.e. a 30% reduction. Based on this outcome, a trial of 120 patients, 60 per group, would have been needed to detect the observed 30% reduction in the proposed composite, from 80% to 50%, with 90% power, assuming a 5% level of statistical significance and a two-sided test. We elected for a larger sample size of 160 patients, 80 per group. In addition, we elected to assess myocardial injury directly using (i) serial plasma release of troponin T in 140 patients, 70 per group; and (ii) serial markers of myocardial oxidative stress in myocardial biopsies in 40 consecutive patients in Bristol, 20 per group, based on the outcome of previous similar work, e.g. [7, 17].

### Randomization and blinding

Patients were assigned immediately prior to surgery to the conventional or hybrid group in a 1:1 ratio using cohort minimization to achieve balance across surgeons (and therefore centres) and type of valve surgery (aortic, mitral or both). Concealed random allocations were generated by computer once the relevant baseline data (information to identify the patient, the surgeon and the type of surgery) had been entered into the system. Intensive care unit consultants and nurses as well as the study participants were masked to the study allocation.

### Statistical methods

Analyses were performed on an intention-to-treat basis and in keeping with a pre-specified statistical analysis plan. Continuous data are summarized as mean standard deviation or median [interquartile range (IQR)] if distributions are skewed. Categorical data are summarized as number (percentage). Outcomes were compared using logistic (binary variables), Cox proportional hazards (time to event variables for secondary outcomes intubation time, intensive care length of stay and hospital stay), or linear mixed model (continuous variables measured at multiple time points) regression, with conventional surgery as the reference group. Model validity was checked using standard methods; if a model fitted poorly, transformations were explored. Outcomes analysed on a logarithmic scale were transformed back to the original scale after analysis and results presented as geometric mean ratios (GMR). See the Supplementary material for further details. All outcomes were adjusted for the stratification variables including: surgical centre, and type of valve surgery. Likelihood ratio tests were used to determine statistical significance, and two-tailed *P*-values < 0.05 were considered statistically significant. A subgroup analysis comparing centre-specific cTnT concentrations was prespecified in the statistical analysis plan. Subgroups were compared, by adding an allocation by centre interaction term into the model. Subgroup-specific effects are reported if the interaction term was statistically significant at the 10% level. All analyses were performed in SAS version 9.3 (SAS Institute Inc, Cary, NC, USA) and Stata version 13.0 (StataCorp LP, College Station, TX, USA).

## RESULTS

### Patient recruitment

**Figure 1:**
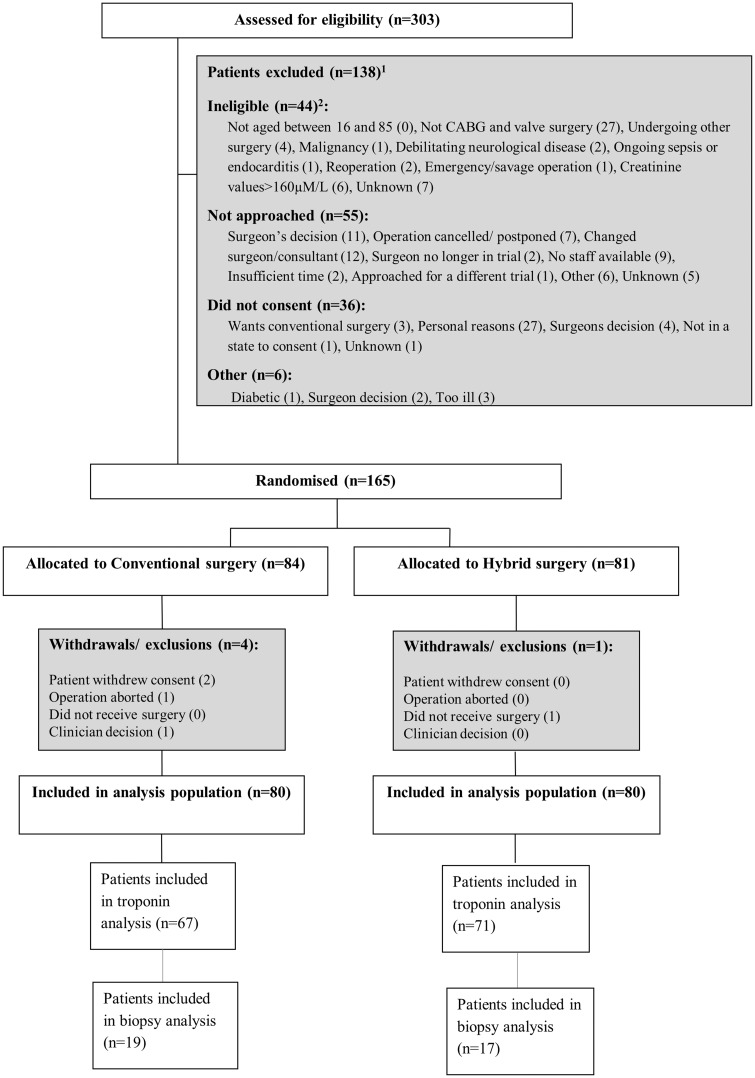
Flow of participants through the trial. Notes: ^1^Reasons for exclusion are only available for the Bristol site. ^2^Some patients were ineligible for more than one reason.

Between 19 March 2008 and 01 July 2012, 165 patients gave written consent and were randomized; 5 were withdrawn prior to surgery, leaving 160 patients who received surgery and were included in analysis population (80 randomized to conventional surgery and 80 to hybrid surgery, Fig. 1). In total, 81 patients were recruited in Bristol, 60 in Kolkata and 19 in Ahmedabad. There were 5 protocol deviations. Serious adverse events were also captured for UK patients (Supplementary Material, Tables SE1–SE3).

### Baseline characteristics

Baseline characteristics were similar between groups (Table 1). The mean age was 66.5 years and 74% of patients were male. The median additive EuroSCORE was 3 for both groups. Intraoperative details are shown in Table 2. Overall, 97 patients had an aortic valve replacement, 52 had a mitral valve replacement or repair and 8 had both (Table 2 and Supplementary Material, Table SE4). All patients received cold blood cardioplegia.

**Table 1:** Baseline characteristics

	Conventional (*n* = 80)		Hybrid (*n* = 80)		Overall (*n* = 160)	
	*n*	%	*n*	%	*n*	%
Demographics						
Age (years) (mean, SD)	66.7	10.12	66.3	9.67	66.5	9.87
Male gender	60	75.0	59	73.8	119	74.4
BMI (mean, SD)	25.5	5.58	25.7	4.97	25.6	5.26
Cardiac history						
NYHA class						
I/Asymptomatic	10	12.5	4	5.0	14	8.8
II	45	56.3	49	61.3	94	58.8
III	22	27.5	25	31.3	47	29.4
IV	3	3.8	2	2.5	5	3.1
Angina class						
No angina	23	28.8	20	25.0	43	26.9
I	8	10.0	14	17.5	22	13.8
II	36	45.0	39	48.8	75	46.9
III	11	13.8	6	7.5	17	10.6
IV	2	2.5	1	1.3	3	1.9
Previous MI	5	6.3	4	5.0	9	5.6
Congestive cardiac failure	7	8.8	7	8.8	14	8.8
Heart rhythm						
Sinus rhythm	63	78.8	66	83.5	129	81.1
Sinus rhythm and heart block	5	6.3	6	7.6	11	6.9
AF/flutter	10	12.5	7	8.9	17	10.7
AF/flutter and heart block	2	2.5	0	0.0	2	1.3
Permanent pacemaker	2	2.5	0	0.0	2	1.3
MI <90 days ago	5	6.3	7	8.8	12	7.5
Baseline LV function						
Good	60	75.0	55	69.6	115	72.3
Moderate	17	21.3	21	26.6	38	23.9
Poor	3	3.8	3	3.8	6	3.77
Coronary disease						
Single	31	38.8	28	35.4	59	37.1
Double	29	36.3	29	36.7	58	36.5
Triple	20	25.0	22	27.8	42	26.4
Left main stem disease	6	7.5	8	10	14	8.8
Other medical history						
Peptic ulceration	5	6.3	2	2.5	7	4.4
Renal failure	4	5.0	2	2.5	6	3.8
Peripheral vascular disease	10	12.5	5	6.3	15	9.4
Severe asthma	2	2.5	2	2.5	4	2.5
Other medical conditions	20	25.0	17	21.3	37	23.1
EuroSCORE	3.0	(2.0, 5.0)	3.0	(1.5, 5.0)	3.0	(2.0, 5.0)

Missing data (conventional, hybrid): BMI, Warfarin, Other drugs: 1 (1, 0). Heart rhythm, IV Nitrates: 1 (0, 1).

BMI: body mass index; MI: myocardial infarction; AF: atrial fibrillation; LV: left ventricular; IV intravenous; SD: standard deviation; NYHA: New York Heart Association.

**Table 2:** Intraoperative details

	Conventional (*n* = 80)		Hybrid (*n* = 80)		Overall (*n* = 160)	
	*n*	%	*n*	%	*n*	%
Valve details						
Valve surgery type:						
Aortic valve	47	61.0	50	62.5	97	61.8
Repaired	1	2.1	0	0.0	1	1.0
Replaced	46	97.9	50	100	96	99.0
Mitral valve	25	32.5	27	33.8	52	33.1
Repaired	12	48.0	14	51.9	26	50.0
Replaced	13	52.0	13	48.1	26	50.0
Both	5	6.5	3	3.8	8	5.1
Both repaired	0	0.0	1	33.3	1	12.5
Both replaced	4	80.0	2	66.7	6	75.0
Mitral repaired, aortic replaced	1	20.0	0	0.0	1	12.5
Coronary details						
No. of coronary grafts (median, IQR)	2.0	(1.0, 2.0)	2.0	(1.0, 2.0)	2.0	(1.0, 2.0)
1	33	41.3	33	41.3	66	41.3
2	29	36.3	30	37.5	59	36.9
3	12	15.0	12	15.0	24	15.0
4	6	7.5	5	6.3	11	6.9
Other procedure^a^	6	7.5	5	6.3	11	6.9

a Two procedures of ablation of atrial fibrillation, 2 tricuspid valve repairs, 1 small left ventricular aneurysm repair, and 1 closure of patent foramen ovale in the conventional group; and 1 closure of patent foramen ovale, 1 aortic root enlargement, 1 removal of left atrial appendix with stapling due to thrombus, 1 ablation of atrial fibrillation and 1 patch closure of a healed ventricular septal defect in the hybrid group.

IQR: interquartile range.

### Primary outcome: composite of complications related to myocardial injury

**Figure 2:**
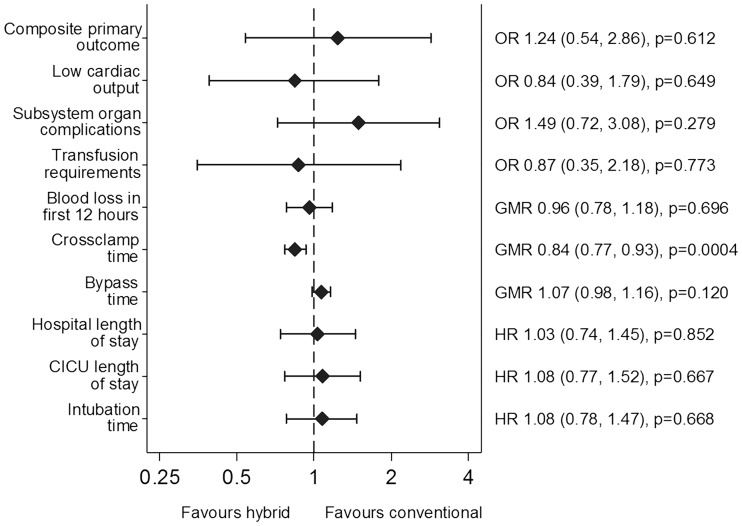
Primary and secondary clinical outcomes. OR and 95% CI for the effect of hybrid versus conventional surgery on the primary and secondary outcomes. CICU: cardiac intensive care unit.

The number of patients experiencing the primary outcome was 64/80 (80.0%) in the conventional surgery group and 67/80 (83.8%) in the hybrid group [odds ratio (OR) 1.24, 95% confidence interval (CI) 0.54–2.86, *P* = 0.61; Fig. 2]. In total, there were 16 in-hospital/30-day deaths (10%), 7 in the conventional group and 9 in the hybrid group (Table 3). Of these, 4/81 (4.9%) occurred in Bristol (2 in the conventional and 2 in the hybrid group) and 12/79 (15.2%) in India (5 in the conventional and 7 in the hybrid group). There was 1 MI (1.3%) in the conventional group. Need for inotropic support >12 h was 50.0% in the conventional group, 55.0% in the hybrid group, while the incidence of new arrhythmia was 46.3% in the conventional group, 32.5% in the hybrid group.

**Table 3:** Primary outcome: cardiac specific composite

	Conventional (*n* = 80)		Hybrid (*n* = 80)		OR (95% CI)	*P*-value
	*n*	%	*n*	%		
Primary outcome							
In-hospital/30-day death	7	8.8	9	11.3		
Postoperative MI	1	1.3	0	0.0		
Arrhythmia	37	46.3	36	45.0		
Cardiac pacing >12 h	33	41.3	26	32.5		
Postoperative inotropic support >12 h^a^	40	50.0	44	55.0		
Composite primary outcome	64	80.0	67	83.8	1.24 (0.54–2.86)	0.612

Missing data (conventional, hybrid): Post-op MI: 2 (1, 1).

a Including dopamine* *≥5 µg/kg/min, dobutamine ≥3 µg/kg/min, adrenaline ≥1 µg/kg/min and any dose of enoximone.

MI: myocardial infarction; OR: odds ratio; CI: confidence interval.

### Secondary outcome: direct myocardial injury

**Figure 3:**
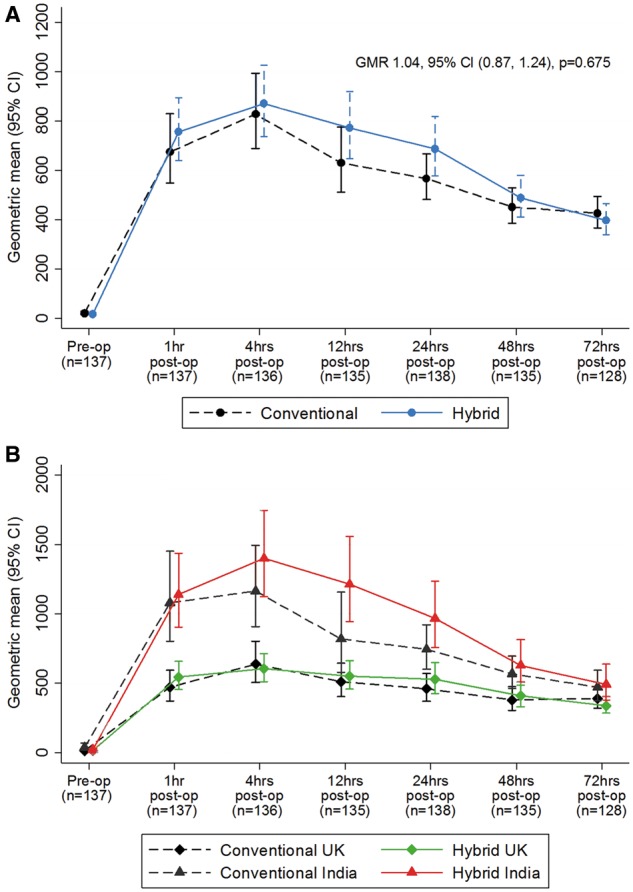
(**A**) Overall troponin concentrations over time by treatment group. Geometric mean (ng/l) and 95% CI at all time points by group, and geometric mean ratio and 95% CI for the effect of hybrid versus conventional surgery on troponin concentration. (**B**) Troponin concentrations over time by treatment group and centre. Geometric mean and 95% CI at all time points by group and centre. Test for treatment by centre interaction, *P* = 0.48. Pre-op: preoperative; Post-op: postoperative.

**Figure 4:**
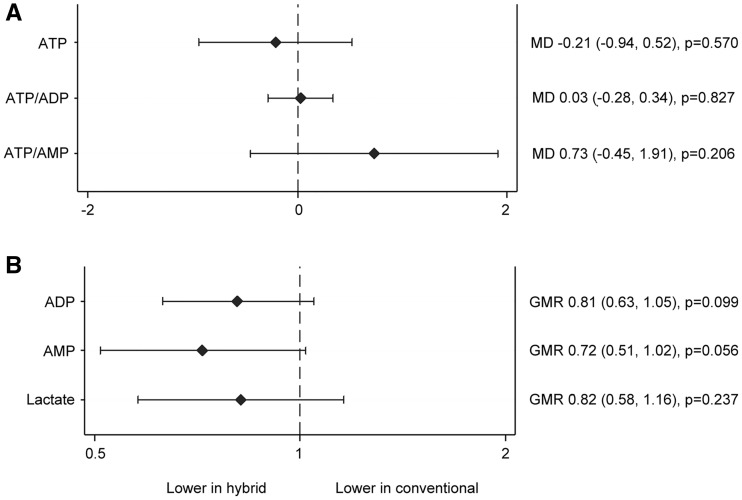
Myocardial metabolic stress outcomes. (**A**) Mean differences (MD) and 95% CI for the effect of hybrid versus conventional surgery on ATP, ATP/ADP and ATP/AMP (nmole/mg wet weight). (**B**) GMR and 95% CI for the effect of hybrid versus conventional surgery on ADP, AMP and Lactate (nmole/mg wet weight). ATP: adenosine triphosphate; ADP: adenosine diphosphate; AMP: adenosine monophosphate.

Cardiac TnT measurements were undertaken in Bristol and Kolkata in 138 patients. Release of cTnT was similar in the 2 groups at all the time points (treatment by time interaction *P* = 0.20, overall estimate of difference between the 2 groups GMR 1.04, 95% CI 0.87–1.24, *P* = 0.68, Fig. 3A, Supplementary Material, Table SE8). There was no difference in the effect of treatment on troponin between the UK and India participants (*P* = 0.48), although higher levels of cTnT release were observed in India compared to Bristol (Fig. 3B). For metabolic stress, levels of adenosine diphosphate and adenosine monophosphate measured in reperfusion biopsies in a subgroup of 36 patients in Bristol were lower compared to baseline in both the hybrid group with GMRs of 0.81 (95% CI 0.63–1.05, *P* = 0.099) and the conventional group 0.72 (95% CI 0.51–1.02, *P* = 0.056). There were no differences between the groups with respect to adenosine triphosphate (ATP), ATP/adenosine diphosphate, ATP/adenosine monophosphate with mean differences of −0.21, 95% CI −0.94 to 0.52, *P* = 0.57; 0.03, 95% CI −0.28 to 0.34, *P* = 0.83 and 0.73, 95% CI −0.45 to 1.91, *P* = 0.21, respectively. Similarly, there was no difference for lactate (GMR 0.82, 95% CI 0.58–1.16, *P* = 0.24, Fig. 4, Supplementary Material, Table SE9).

### Operative details and other clinical outcomes

Operative details are shown in Table 4. Duration of CA was reduced from a median of 98 min (IQR 79–135) in the conventional group to 89 min (IQR 63–118) in the hybrid group and this was statistically significant (GMR 0.84, 95% CI 0.77–0.93, *P* = 0.0004). CPB time was 7% longer in the hybrid group (GMR 1.07, 95% CI 0.98–1.16, *P* = 0.12). Operation time was 4% longer in the hybrid group (*P* = 0.2). Descriptive only key secondary clinical outcome including pulmonary, infective, renal and neurological complications are shown in Table 4. The incidence of stroke was 1.3% vs 2.6% in the conventional versus hybrid group respectively.

**Table 4:** Secondary clinical outcome

	Conventional (*n* = 80)		Hybrid (*n* = 80)		OR (95% CI)	*P*-value
	*n*	%	*n*	%		
Intraoperative outcome						
Operation time (median, IQR)	288	(220, 403)	300	(245, 405)	GMR=1.18 (0.94–1.21)	0.18
CPB time (min) (median, IQR)	142	(105, 195)	153	(115, 233)	GMR=1.07 (0.98–1.16)	0.12
CA time (min) (median, IQR)	98	(79, 135)	89	(63, 118)	GMR=0.84 (0.77–0.93)	0.0004
Other postoperative outcome						
Reintubation	4	5.2	6	7.8		
Tracheostomy	4	5.2	3	4		
Septicaemia	2	2.6	2	2.6		
Sternotomy infection	3	4	1	1.3		
Renal failure with dialysis	5	5.2	6	7.8		
Stroke	1	1.3	2	2.6		
Transient ischaemic attack	0	0.0	2	2.6		

OR: odds ratio; CI: confidence interval; IQR: interquartile range; GMR: geometric mean ratio; CPB: cardiopulmonary bypass; CA: cardioplegic arrest.

There was no difference between groups for low-cardiac output (29/80, 36.3% vs 26/80, 32.5%, OR 0.84, 95% CI 0.39–1.79, *P* = 0.65), transfusion requirement (66/80, 82.5% vs 65/80, 82.3%, OR 0.87, 95% CI 0.35–2.18, *P* = 0.77), blood loss (median 363 vs 400 ml, GMR 0.96, 95% CI 0.78–1.18, *P* = 0.70), combined non-cardiac subsystem organ complication (44/50, 55.0% vs 51/50, 63.8%, OR 1.49, 95% CI 0.72–3.08, *P* = 0.28), serious adverse events, intubation time, cardiac intensive care unit length of stay and hospital stay being also similar between the 2 groups (Fig. 2, Supplementary Material, Tables SE5–SE7, Fig. SE1).

## DISCUSSION

This is the first trial to evaluate the hybrid procedure in patients undergoing combined coronary and valve surgery. The incidence of the composite primary endpoint was 80% in the conventional group of this trial. This confirms methodologically the excellent predictive value of our preliminary pilot study, in which the frequency of the same composite was also 80%. However for the hybrid group a 50% composite event rate was assumed but an 83.8% was observed, suggesting that the experimental group did not fulfil the protocol assumptions. Accordingly, the trial suggests that, although the CA time was statistically shortened (9 min) in the hybrid group this difference had no effect on measures of myocardial injury including the selected composite as well as the release of cTnT and markers of metabolic stress.

The trial confirms that the hybrid procedure is surgically feasible and reproducible; 12 surgeons participated across the 3 centres and there was only 1 (1.2%) cross-over from hybrid to conventional surgery. It also suggests that the hybrid procedure is safe; health outcomes were similar to the conventional technique overall and across the 3 centres, although the incidence of mortality was higher in the Indian centres (15.2%) compared to the UK centre (4.9%), probably reflecting the geographical differences in patient characteristics, health outcome, and service provision. The health outcome of the Bristol centre is in keeping with current North American and European standards populations [23, 24].

Shortening of CA time by 9 min was insufficient to demonstrate a clinical benefit. The saving of only 9 min was probably triggered by a high proportion of single or double-vessel coronary disease recruited (74%), leading to 78% of patients receiving 1 or 2 bypass grafts. Indeed, the amount of CA time saved increased with the number of bypass grafts performed (difference in geometric means 6, 15 and 26 min for operations involving 1, 2 and 3 or more bypass grafts, respectively), but no subanalysis was undertaken to assess if longer CA time savings were associated with myocardial injury in keeping with the predefined statistical analysis plan.

The concept of shortening CA time to minimize myocardial injury is not new. Perrault *et al.* [25] suggested that on-pump beating heart coronary revascularization in patients with poor-left ventricular function and acute myocardial ischaemia eliminated CA related myocardial ischaemia and its negative consequences. However, in a randomized controlled trial in 80 patients randomized to conventional versus on-pump beating heart coronary surgery, we showed comparable results [22].

The use of on-pump beating heart coronary surgery in patients with valve disease has been reported only in anecdotal studies [26, 27]. Our trial provides information that is not subject to the selection bias typical of retrospective, small observational studies.

The impact of the hybrid procedure on the clinical components of the composite end-point suggests that this was a negative trial. We selected these components due to their direct relation with myocardial injury. This is in keeping with the evidence by others suggesting that a CA time >75 min is associated with a higher risk of cardiac death and major adverse cardiac events and that high levels of cTnT are associated with major adverse cardiac events [24, 28]. The negative outcome of the trial and the validity of the selected composite are confirmed beyond any doubt by the direct measures of myocardial injury we used, which also did not show differences and included release of cTnT and markers myocardial metabolic stress. cTnT release in the hybrid group confirmed that 9 min only of CA time saving is not sufficient to reduce myocardial injury in keeping with the lack of difference in levels of ATP, lactate and adenosine triphosphate (ATD)/adenosine diphosphate ratio in myocardial biopsies.

In both groups, the median CA time was higher than the critical threshold of 75 min suggested by Liakopoulos *et al.* [28]. Of note, Nesher *et al.* [24] suggest a strong association between levels of cTnT exceeding 1.3 μg/l and major adverse cardiac events. Our data support this observation as the geometric mean of the maximum cTnT release was 1024 ng/l for those who experienced the primary outcome and 769 ng/l for those who did not.

The study has limitations. There were some protocol deviations and 5 randomized patients were excluded from the analysis population. Including all valve pathologies provided an inclusive but potentially heterogeneous population. Follow-up was also limited and quality of life was not assessed. In addition, it may be argued that the 5 components of the composite differ in quantitative clinical importance i.e. a death or a large MI impact more negatively than an arrhythmic event or need for inotropes or for pacing. However, these components were selected as they are all qualitatively clinical reflections of myocardial injury, regardless of their quantitative impact.

## CONCLUSION

The hybrid procedure was surgically feasible, reproducible and safe. The statistically significant reduction in CA time of 9 min was not clinically meaningful as clinical and biochemical measures of myocardial injury were similar between groups. The hybrid procedure is not superior to conventional technique.

## SUPPLEMENTARY MATERIAL

Supplementary material is available at *EJCTS* online.

## Supplementary Material

Supplementary Data
