# Supplementary material for: Shortening cardioplegic arrest time in patients undergoing combined coronary and valve surgery: results from a multicentre randomized controlled trial: the SCAT trial
Source: Eur J Cardiothorac Surg. 2017 Apr 24;52(2):288–96. doi: 10.1093/ejcts/ezx087 (PMC5848808; doi:10.1093/ejcts/ezx087)
Supplement: Supplementary Data [file ezx087_supp.docx]

**Supplementary data**

**Statistical Methods—Further Details**

When analyzing myocardial troponin T (cTnT) release, the baseline and posttreatment values were modelled jointly to avoid having to exclude or impute cases with missing baseline measures. Similarly, when analysing measures of ischaemic stress assessed in the myocardial biopsies, the measurement taken 5 minutes following CPB institution (control biopsy) was modelled jointly with the reperfusion biopsy taken 10 minutes after releasing the cross clamp.

When analysing intubation time, and length of stay outcomes, any patient who died prior to the event was treated as a censored observation.

**Table E1 Protocol deviations**

|  | **Randomised to Conventional group (n=84)** | | **Randomised to Hybrid group (n=81)** | | **Overall (n=165)** | |
| --- | --- | --- | --- | --- | --- | --- |
|  | **n** | **%** | **n** | **%** | **n** | **%** |
| Any protocol deviations | 8 | 9.5% | 2 | 2.5% | 10 | 6.1% |
| Randomised but not treated | 4 | 4.8% | 1 | 1.2% | 5 | 3.0% |
|  | **Treated in Conventional group (n=80)** | | **Treated in Hybrid group (n=80)** | | **Overall (n=160)** | |
|  | **n** | **%** | **n** | **%** | **n** | **%** |
| Did not meet eligibility criteria | 1 | 1.3% | 0 | 0.0% | 1 | 0.6% |
| Did not receive allocated treatment | 0 | 0.0% | 1 | 1.3% | 1 | 0.6% |
| Only received CABG | 3 | 3.8% | 0 | 0.0% | 3 | 1.9% |
| Only received valve | 0 | 0.0% | 0 | 0.0% | 0 | 0.0% |

**Table E2 Details of protocol deviations where available**

| **Allocation** | **Centre** | **Further details** |
| --- | --- | --- |
| *Randomised but not treated* | | |
| Conventional | A | Patient had porcelain aorta, operation did not proceed |
| Conventional | B | Patient consented and withdrew prior to surgery |
| Conventional | B | Only received AVR only |
| Conventional | B | Patient withdrew consent so data not collected |
| Hybrid | C | During Pre-operative period Pulmonary Artery pressure was found to be higher than systemic pressure, with Right Ventricular pressure being 110mmHg. In view of these findings surgery was not carried out given the extremely high risk associated with this condition. |
| *Did not meet eligibility criteria* | | |
| Conventional | B | Creatinine level was 176 µm/l |
| *Did not receive allocated treatment* | | |
| Hybrid | A | The patient had conventional surgery as the surgeon was not comfortable with the available cardiac stabiliser |
| *Only received CABG* | | |
| Conventional | A | Reason was not given on study case report form |
| Conventional | A | Porcelain aorta. |
| Conventional | A | Intra-operative echocardiogram showing only mild valvular disease. |

**Table E3 Withdrawals**

|  | | **Randomised to Conventional surgery (n=84)** | | **Randomised to Hybrid surgery (n=81)** | | **Overall (n=165)** | | |  |
| --- | --- | --- | --- | --- | --- | --- | --- | --- | --- |
|  | | **n** | **%** | **n** | **%** | | **n** | **%** | |
| Any withdrawal | | 4 | 5% | 1 | 1% | | 5 | 3% | |
| *Time of withdrawal* | Pre-op | 2 |  | 1 |  | | 3 |  | |
|  | Intra-op | 2 |  | 0 |  | | 2 |  | |
| *Decision taken by* | Patient | 2 |  | 0 |  | | 2 |  | |
|  | Clinician | 2 |  | 1 |  | | 3 |  | |
| *Reason for withdrawal* | Surgery not needed | 0 |  | 1 |  | | 1 |  | |
|  | Operation aborted | 1 |  | 0 |  | | 1 |  | |
|  | Only CABG needed | 1 |  | 0 |  | | 1 |  | |
|  | Reason unknown | 2 |  | 0 |  | | 2 |  | |

**Table E4 Intra-operative characteristics, additional information**

|  | | **Conventional (n=80)** | | **Hybrid**  **(n=80)** | | **Overall**  **(n=160)** | |
| --- | --- | --- | --- | --- | --- | --- | --- |
|  | | **n** | **%** | **n** | **%** | **n** | **%** |
| ~~Operation time (mins) (Median, IQR)~~ | | ~~288~~ | ~~(220, 403)~~ | ~~300~~ | ~~(245, 405)~~ | ~~297~~ | ~~(230, 403)~~ |
| Lowest HCT (Mean, SD) | | 23.5 | 4.12 | 22.4 | 3.77 | 23.0 | 3.97 |
| Lowest temperature (°C) (Median, IQR) | | 30.0 | (28.0, 32.0) | 30.2 | (28.0, 32.0) | 30.0 | (28.0, 32.0) |
| No sinus rhythm on cross clamp removal | | 36 | *46.3%* | 21 | *26.5%* | 57 | *35.6%* |
|  | *AV Block* | *19* | *24.4%* | *14* | *17.6%* | *33* | *20.8%* |
|  | *Atrial fibrillation* | *8* | *10.3%* | *3* | *3.8%* | *11* | *7.0%* |
|  | *Ventricular fibrillation* | *7* | *9.0%* | *3* | *3.8%* | *10* | *6.3%* |
|  | *Junctional rhythm* | *2* | *2.6%* | *1* | *1.3%* | *3* | *1.9%* |
| DC Cardioversion | | 16 | 20.0% | 10 | 12.5% | 26 | 16.3% |
| Intra-operative IABP | | 5 | 7.1% | 4 | 5.4% | 9 | 6.3% |
|  | Duration (hrs) (Median, IQR) | 3.0 | (3.0, 5.0) | 19.8 | (2.8, 65.5) | 3.5 | (3.0, 36.0) |
| Intra-operative transfusion requirements^1^ | |  |  |  |  |  |  |
|  | RBC (Products, Patients) | 109 | 42 | 119 | 46 | 228 | 88 |
|  | FFP (Products, Patients) | 16 | 7 | 25 | 12 | 41 | 19 |
|  | Platelets (Products, Patients) | 59 | 22 | 81 | 30 | 140 | 52 |

|  |  |  |  |  |  |  |
| --- | --- | --- | --- | --- | --- | --- |

^1^ Data on all transfusions given, either intraoperatively or postoperatively, are reported in Table E5.*HCT* haematocrit; *IQR* inter-quartile range; *AV* Atrio-ventricular; *DC* Direct Current; *RBC* Red Blood Cells; *FFP* Fresh Frozen Plasma; IABP intra-aortic balloon pump; IQR interquartile range; GTN glyceryl trinitrate; SNP sodium nitroprusside. Missing data (conventional, hybrid): Lowest HCT, Lowest core temperature, Rhythm on clamp removal: 2 (2, 0).

**Table E5 secondary outcomes**

|  | | **Conventional (n=80)** | | **Hybrid (n=80)** | | **OR/HR**  **GMR**  **(95% CI)** | **P-value** |
| --- | --- | --- | --- | --- | --- | --- | --- |
|  | | **n** | **%** | **n** | **%** |  |  |
| **INTRA-OP OUTCOMES** | |  |  |  |  |  |  |
| ~~Duration of CPB (mins) (Median, IQR)~~ | | ~~142~~ | ~~(105, 195)~~ | ~~153~~ | ~~(115, 233)~~ | ~~GMR=1.07~~  ~~(0.98, 1.16)~~ | ~~0.120~~ |
| ~~Duration of CA (mins) (Median, IQR)~~ | | ~~98~~ | ~~(79, 135)~~ | ~~89~~ | ~~(63, 118)~~ | ~~GMR=0.84~~  ~~(0.77, 0.93)~~ | ~~0.0004~~ |
| **INTRA-OP/POST-OP TRANSFUSION** | |  |  |  |  |  |  |
| Any transfusion requirement | | 66 | 82.5% | 65 | 82.3% | OR= 0.87  (0.35, 2.18) | 0.773 |
|  | *Red blood cells (units, patients)* | *222* | *65* | *249* | *62* |  |  |
|  | *Fresh frozen plasma (units, patients)* | *72* | *17* | *80* | *23* |  |  |
|  | *Platelets (units, patients)* | *120* | *28* | *143* | *33* |  |  |
|  | *Cryoprecipitate (units, patients)* | *4* | *2* | *10* | *3* |  |  |
| **POST-OP COMPLICATIONS** | |  |  |  |  |  |  |
| Any subsystem organ complication | | 44 | 55.0% | 51 | 63.8% | OR= 1.49  (0.72, 3.08) | 0.279 |
|  | *Cardiovascular* | *39* | *48.8%* | *39* | *48.8%* |  |  |
|  | *Pulmonary* | *9* | *11.3%* | *10* | *12.5%* |  |  |
|  | *Infective* | *13* | *16.3%* | *14* | *17.5%* |  |  |
|  | *Renal* | *5* | *6.3%* | *6* | *7.5%* |  |  |
|  | *Gastrointestinal* | *2* | *2.5%* | *5* | *6.3%* |  |  |
|  | *Neurological* | *2* | *2.5%* | *8* | *10.0%* |  |  |
| Low cardiac output^1^ | | 29 | 36.3% | 26 | 32.5% | OR= 0.84  (0.39, 1.79) | 0.649 |
| Blood loss (12 hours post-op; ml) (Median, IQR) | | 363 | (228, 615) | 400 | (245, 600) | GMR= 0.96  (0.78, 1.18) | 0.696 |
| Extubation time (hours) (Median, IQR)^2^ | | 16.5 | (7.6, 24.9) | 17.1 | (7.1, 51.3) | HR=0.93  (0.68, 1.29) | 0.668 |
| ICU stay (hours) (Median, IQR)^3^ | | 51.3 | (22.7, 121.0) | 67.5 | (23.5, 122.2) | HR=0.93  (0.66,1.30) | 0.667 |
| Hospital stay (days) (Median, IQR) | | 10.0 | (7.0, 14.0) | 9.0 | (8.0, 13.0) | HR=0.97  (0.69, 1.35) | 0.852 |

^1^ Defined as adrenaline, dopamine, dobutamine or enoximone (at a dose of ≥ 5ug/kg/min) or IABP given consecutively to patient for 3 hours or more; ^2^Includes any re-intubation (number of re-intubations in conventional group=7, in hybrid group=8); ^3^ Includes any readmission (number of readmissions in conventional group=1, in hybrid group=0); ICU= intensive care unit; IABP= intra-aortic balloon pump. Missing data (conventional, hybrid): Duration of cardiopulmonary bypass, Duration of aortic cross clamp: 2 (2, 0). Transfusion requirements: 1 (0, 1).

**Figure E1 Intubation time (A), intensive care (B) and hospital stay (C) by group**

1. **Intubation time (B) Stay in cardiac intensive care**

1. **Stay in hospital**

**Table E6 In-hospital adverse events**

|  | | **Randomised to**  **Conventional surgery**  **(n=80)** | | | **Randomised to**  **Hybrid surgery**  **(n=80)** | | | **Overall**  **(n=160)** | | |
| --- | --- | --- | --- | --- | --- | --- | --- | --- | --- | --- |
|  | | **Events** | **Patients** | **%^1^** | **Events** | **Patients** | **%^1^** | **Events** | **Patients** | **%^1^** |
| **CARDIAC COMPLICATIONS** | | 49 | 39 | 49% | 43 | 39 | 49% | 92 | 78 | 49% |
| Post-operative MI | | 1 | 1 | 1% | 0 | 0 | 0% | 1 | 1 | 1% |
| Any arrhythmias | | 42 | 37 | 46% | 36 | 36 | 45% | 78 | 73 | 46% |
|  | *SVT/AF* | *39* | *37* | *46%* | *35* | *35* | *44%* | *74* | *72* | *45%* |
|  | *VF/VT* | *3* | *3* | *4%* | *0* | *0* | *0%* | *3* | *3* | *2%* |
|  | *Other* | *0* | *0* | *0%* | *1* | *1* | *1%* | *1* | *1* | *1%* |
| **PULMONARY COMPLICATIONS** | | 15 | 9 | 11% | 14 | 10 | 13% | 29 | 19 | 12% |
| Re-intubation | | 4 | 4 | 5% | 6 | 6 | 8% | 10 | 10 | 6% |
| Tracheostomy | | 4 | 4 | 5% | 3 | 3 | 4% | 7 | 7 | 4% |
| Mask CPAP | | 6 | 6 | 8% | 5 | 5 | 6% | 11 | 11 | 7% |
| ARDS | | 1 | 1 | 1% | 0 | 0 | 0% | 1 | 1 | 1% |
| **INFECTIVE COMPLICATIONS** | | 16 | 13 | 16% | 19 | 14 | 18% | 35 | 27 | 17% |
| Septicaemia | | 2 | 2 | 3% | 2 | 2 | 3% | 4 | 4 | 3% |
| Chest infection | | 11 | 10 | 13% | 14 | 14 | 18% | 25 | 24 | 15% |
| Sternotomy infection | | 3 | 3 | 4% | 3 | 1 | 1% | 6 | 4 | 3% |
| **RENAL COMPLICATIONS** | | 5 | 5 | 6% | 7 | 6 | 8% | 12 | 11 | 7% |
| Need for haemofiltration/dialysis | | 5 | 5 | 6% | 7 | 6 | 8% | 12 | 11 | 7% |
| **GI COMPLICATIONS** | | 2 | 2 | 3% | 6 | 5 | 6% | 8 | 7 | 4% |
| Peptic ulcer/ GI bleed/ Perforation | | 1 | 1 | 1% | 3 | 3 | 4% | 4 | 4 | 3% |
| Other GI (e.g. bowel obstruction) | | 1 | 1 | 1% | 3 | 3 | 4% | 4 | 4 | 3% |
| **CEREBRAL COMPLICATIONS** | | 2 | 2 | 3% | 9 | 8 | 10% | 11 | 10 | 6% |
| Stroke | | 1 | 1 | 1% | 2 | 2 | 3% | 3 | 3 | 2% |
| Transient Ischaemic Attack | | 0 | 0 | 0% | 2 | 2 | 3% | 2 | 2 | 1% |
| Coma or confusion state | | 2 | 2 | 3% | 5 | 4 | 5% | 7 | 6 | 4% |
| **OTHER ADVERSE EVENT^2^** | | 5 | 3 | 4% | 3 | 3 | 4% | 8 | 6 | 4% |

^1^ Percentage of patients^; 2^ One patient in the conventional group had a pleural effusion drained associated with surgical rewiring, 1 patient developed a left lung consolidation and an episode of ileus, and 1 had sternal oozing, and cellulitis in both legs. In the hybrid group 1 patient suffered a grand mal seizure, 1 had a convulsion, and 1 patient had haemodynamic instability.

Note. There was one patient who crossed over from hybrid to conventional surgery. This patient experienced a stroke as adverse event.

*MI* myocardial infarction; *SVT* supraventricular tachycardia; *AF* atrial fibrillation; *VT* ventricular tachycardia; *VF* ventricular fibrillation; *CPAP* continuous positive airway pressure; *ARDS* Acute respiratory distress syndrome; *GI* gastrointestinal. Missing data (conventional, hybrid): Post-operative MI: 2 (1, 1).

**Table E7 Other Post-operative characteristics**

|  | | **Conventional group (n=80)** | | **Hybrid group (n=80)** | | **Overall (n=160)** | |
| --- | --- | --- | --- | --- | --- | --- | --- |
|  | | **n** | **%** | **n** | **%** | **n** | **%** |
| Post-operative IABP | | 5 | 6.3% | 6 | 7.5% | 11 | 6.9% |
|  | Duration (hours) (Median, IQR) | 60.0 | (43.0, 157.0) | 40.5 | (23.0, 61.0) | 57.0 | (23.0, 67.0) |
| Post-operative transfusion (first 24 hours) | | |  |  |  |  |  |
|  | Red blood cells (products, patients) | 99 | 50 | 123 | 53 | 222 | 103 |
|  | Fresh frozen plasma (products, patients) | 31 | 11 | 33 | 15 | 64 | 26 |
|  | Platelets (products, patients) | 18 | 9 | 42 | 19 | 60 | 28 |
|  | Cryoprecipitate (products, patients) | 2 | 1 | 2 | 1 | 4 | 2 |
| Chest reopened | | 14 | 17.5% | 9 | 11.3% | 23 | 14.4% |
|  | *Bleeding* | *10* |  | *6* |  | *16* |  |
|  | *Low cardiac output* | *4* |  | *2* |  | *6* |  |
|  | *Mediastinitis/rewiring* | *0* |  | *1* |  | *1* |  |
|  |  |  |  |  |  |  |  |

HDU high dependency unit, IQR interquartile range, IABP intra-aortic balloon pump, GTN glyceryl trinitrate, SNP sodium nitroprusside

**Table E8 Troponin**

|  | **Randomised to Conventional surgery (n=67)** | | **Randomised to**  **Hybrid surgery**  **(n=71)** | | **GMR**  **(95% CI)** | **P-value** |
| --- | --- | --- | --- | --- | --- | --- |
|  | **Median** | **IQR** | **Median** | **IQR** |  |  |
| Pre-op | 13.9 | (10.4, 26.9) | 14.4 | (10.3, 27.0) |  |  |
| 1 hour post-op | 614.6 | (421, 1130) | 756.7 | (433, 1360) |  |  |
| 4 hour post-op | 861.1 | (518, 1425) | 847.7 | (498, 1422) |  |  |
| 12 hour post-op | 652.8 | (403, 1029) | 778.3 | (408, 1209) |  |  |
| 24 hour post-op | 554.9 | (370, 995) | 666.0 | (382, 1116) |  |  |
| 48 hour post-op | 471.3 | (303, 714) | 430.1 | (298, 746) |  |  |
| 72 hour post-op | 411.4 | (286, 613) | 356.7 | (253, 555) |  |  |
| Test for treatment*time interaction |  |  |  |  |  | 0.183 |
| Overall estimate of treatment effect |  |  |  |  | 1.04  (0.87, 1.24) | 0.675 |

Data available for 138 patients, though not all patients have data at all time points.

*Pre-op* pre-operative; *post-op* post-operative; *IQR* interquartile range; *GMR* geometric mean ratio; *CI* confidence interval; *UK* United Kingdom; *CABG* coronary artery bypass graft

Missing data at each time point (conventional, hybrid); pre-op (0, 1) 1 hour (0, 1), 4 hours (0, 2), 12 hours (2, 1), 24 hours (0, 0), 48 hours (0, 3), 72 hours (4,6)

**Table E9 Reperfusion Biopsies**

|  | **Randomised to Conventional surgery (n=19)** | | **Randomised to Hybrid surgery (n=17)** | | **MD/GMR**  **(95% CI)** | **P-value** |
| --- | --- | --- | --- | --- | --- | --- |
|  | **Median** | **IQR** | **Median** | **IQR** |  |  |
| ATP | 2.35 | (1.65, 3.77) | 2.73 | (1.60, 3.35) | MD= -0.21  (-0.94, 0.52) | 0.570 |
| ADP | 2.06 | (1.47, 2.44) | 1.73 | (1.00, 2.35) | GMR= 0.81 (0.63, 1.05) | 0.099 |
| AMP | 0.94 | (0.40, 1.04) | 0.70 | (0.41, 0.97) | GMR= 0.72 (0.51, 1.02) | 0.056 |
| ATP/ADP | 1.35 | (1.06, 1.58) | 1.51 | (1.22, 1.81) | MD= 0.03  (-0.28, 0.34) | 0.827 |
| ATP/AMP | 3.13 | (2.26, 4.20) | 4.17 | (2.98, 5.45) | MD= 0.73  (-0.45, 1.91) | 0.206 |
| Lactate | 5.53 | (4.08, 8.70) | 4.63 | (2.88, 8.72) | GMR= 0.82 (0.58, 1.16) | 0.237 |

Three patients (2 conventional, 1 hybrid) are omitted from the table due to missing/inaccurate reperfusion biopsies. However, they still contributed to the analyses as they had a successful control (baseline) biopsy taken.

*ATP* adenosine triphosphate*; ADP* adenosine diphosphate*; AMP* adenosine monophosphate*; IQR* interquartile range; *GMR* geometric mean ratio; *MD* mean difference; *CI* confidence interval;
